# Adsorption and Bulk Assembly of Quaternized Hydroxyethylcellulose–Anionic Surfactant Complexes on Negatively Charged Substrates

**DOI:** 10.3390/polym17020207

**Published:** 2025-01-15

**Authors:** Maud Nivard, Francisco Ortega, Ramón G. Rubio, Eduardo Guzmán

**Affiliations:** 1 Ecole Nationale Supérieure de Chimie de Lille (ENSCL), Cité Scientifique—Bât. C7, Avenue Mendeleïev—CS 90108, 59652 Villeneuve D’Ascq Cedex, France; maud.nivard@enscl.centralelille.fr; 2 Departamento de Química Física, Facultad de Ciencias Químicas, Universidad Complutense de Madrid, Ciudad Universitaria, Plaza de la Ciencias s/n, 28040 Madrid, Spain; fortega@quim.ucm.es (F.O.); rgrubio@quim.ucm.es (R.G.R.); 3 Instituto Pluridisciplinar, Universidad Complutense de Madrid, Paseo Juan XXIII 1, 28040 Madrid, Spain

**Keywords:** adsorption, cosmetics, polyelectrolyte–surfactant mixtures, self-assembly

## Abstract

This study examines the adsorption and bulk assembly behaviour of quaternized hydroxyethylcellulose ethoxylate (QHECE)–sodium dodecyl sulphate (SDS) complexes on negatively charged substrates. Due to its quaternized structure, QHECE, which is used in several industries, including cosmetics, exhibits enhanced electrostatic interactions. The phase behaviour and adsorption mechanisms of QHECE–SDS complexes are investigated using model substrates that mimic the wettability and surface charge of damaged hair fibres. Two preparation methodologies, *high-concentration mixing* and *gradient-free mixing*, were employed to examine their impact on the complex equilibrium, phase behaviour, and adsorption properties of the complexes. The measurements of turbidity, electrophoretic mobility, and conductivity demonstrate the existence of nonequilibrium dynamics during the mixing process, which exert a significant influence on the structural and functional characteristics of the complexes. The quartz crystal microbalance with dissipation monitoring (QCM-D) was employed to investigate the adsorption of the complexes onto the substrates. The results demonstrated the critical role of intermediate SDS concentrations in enhancing deposition. The findings emphasise the importance of formulation and preparation protocols in designing stable, high-performance cosmetic products. This research advances our understanding of polyelectrolyte–surfactant interactions and provides insights into optimising QHECE-based formulations.

## 1. Introduction

Hydroxyethylcellulose (HEC) is a water-soluble polymer derived from cellulose, widely utilised for its cost-effectiveness, non-toxic nature, and environmentally friendly character. It has garnered significant attention across various industries, including food, pharmaceuticals, cosmetics, and paper production, due to its multifunctional properties as a binder, thickening agent, stabiliser, suspension agent, and water retention aid [1,2,3,4,5]. HEC is particularly valued in cosmetic formulations for its ability to enhance product texture and stability. Its thickening and stabilising effects make it a key ingredient for creams, lotions, and gels, where consistent viscosity and smooth application are crucial. Additionally, HEC facilitates the formation of stable emulsions and suspensions, ensuring the even distribution of active ingredients and pigments. Moreover, its film-forming capabilities further contribute to moisture retention and conditions providing protective barriers on the skin or hair [6,7,8].

The quaternisation of HEC, which introduces cationic sites along its molecular structure, significantly enhances its solubility in water solubility and increases its hydrophilicity. It must be stressed that the quaternisation method allows one to tune the density of the QHEC chains [9]. This modification improves the polymer’s electrostatic interactions with negatively charged molecules and surfaces. This enhanced functionality expands the application range of quaternized HEC (QHEC), making it a promising candidate for developing functionalised surfaces, antimicrobial films, and drug delivery systems. In the cosmetics industry, QHEC, often called Polyquaternium-10 or JR400, plays a crucial role as a conditioning agent for hair and skin. Its cationic nature enhances the deposition and retention of conditioning layers, improving smoothness, softness, and the overall sensory experience. This is especially true in shampoos and conditioners, where QHEC interacts with anionic surfactants to form conditioning layers that persist on hair even after rinsing, providing long-lasting conditioning benefits [6,10,11,12]. The increased solubility and binding capacity of QHEC have been leveraged in applications such as enhanced oil recovery from geological reservoirs. Its ability to interact with negatively charged surfaces aids in the efficient displacement of oil, further showcasing the polymer’s versatility across different sectors [13,14,15]. The quaternisation also enhances the ability of cellulose-based polymers to interact with oppositely charged surfactants, including SDS molecules, which can expand the application range of this type of polymers [16]. Moreover, quaternisation has been shown to improve the viscosity in relation to an HEC solution, even at low concentrations, indicating its potential as a superior thickening agent in applications requiring enhanced viscosity and stability [17,18].

This study focuses on the interaction between quaternized hydroxyethylcellulose ethoxylate (QHECE) and sodium dodecyl sulphate (SDS) in model washing solutions. Specifically, it examines the influence of SDS on the deposition of QHECE complexes onto negatively charged model surfaces. These model surfaces are designed to mimic the charge density and contact angle of damaged hair fibres, providing valuable insights into the conditioning performance of cosmetic formulations. The effectiveness of conditioning products is known to largely depend on the deposition of polyelectrolyte–surfactant complexes and other species onto the surface of damaged hair fibres [6,11,19]. Damaged hair fibres, especially those exposed to weathering or chemical treatments, often exhibit a high surface density of sulfonate groups, making them particularly receptive to cationic conditioning agents. However, the complex chemical structure and high surface roughness of real hair fibres make it challenging to study the deposition process in situ on hair fibres [20,21,22]. To address this challenge, negatively charged flat surfaces, such as gold surfaces modified with ω-mercapto-alkyl-sulfonic acid or silicon wafers enriched with silanol groups, have been extensively used as model substrates. These surfaces effectively replicate some key attributes of damaged hair fibres, allowing for a more precise investigation of the fundamental physicochemical mechanisms that govern the deposition of conditioning agents [6,11,23,24,25,26]. In this work, we study the adsorption behaviour of binary QHECE–SDS mixtures with a fixed QHECE concentration and different SDS ones on these model surfaces. Furthermore, the self-assembly of polyelectrolyte–surfactant complexes in bulk solutions is characterised. This aspect is crucial because different studies have shown that the mixing procedures influence the equilibration of polyelectrolyte–surfactant mixtures in solution [27,28,29,30,31,32,33,34]. In fact, according to the literature, polyelectrolyte–surfactant interactions are highly sensitive to the surfactant concentration and mixing dynamics, leading to a range of structural behaviours in both bulk solution and at interfaces [29]. Based on the above, the effect of ageing in the samples prepared by two different mixing approaches will be explored, and the adsorption on the negatively charged surfaces of the aged sample will be analysed. While previous studies have investigated the phase behaviour and adsorption characteristics of polyelectrolyte–surfactant complexes, the interplay between mixing dynamics and complex formation is not fully understood. Through this study, it is expected to shed light on the critical role of mixing dynamics in governing the structural and functional properties of these complexes. This may provide a more realistic approach to what happens during storage after the production of shampoo formulations.

## 2. Materials and Methods

### 2.1. Chemicals

**Figure 1:**
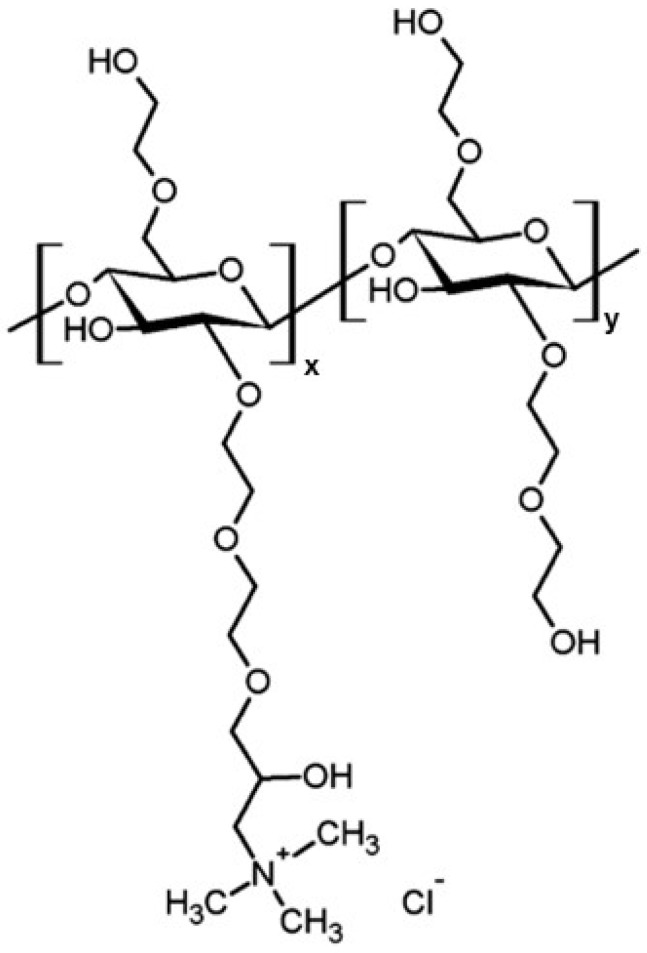
Molecular formular for QHECE. Notice that the used QHECE is a random copolymer containing quaternized monomers and non-quaternized monomers in a molar ratio of 3:7. Therefore, the subindex x and y assume values of 0.3 and 0.7, respectively. Adapted from Roa et al. [35], Copyright (2023), with permission from Elsevier.

Hydroxyethylcellulose ethoxylate, quaternized, QHECE (see Figure 1 for molecular formula), with an average molecular weight of 400 kDa and a quaternisation degree around 30%, was purchased from Sigma-Aldrich (Saint Louis, MO, USA) [35]. Sodium dodecyl sulphate, SDS, with a purity >99%, was supplied by Fisher (Waltman, MA, USA). All the chemicals were used as received without further purification.

The solutions were prepared with ultrapure deionised water of Milli-Q grade, produced using an AquaMAX™-Ultra 370 Series multi-cartridge purification system from Young Lin Instrument Co., Ltd. (Anyang-si, Republic of Korea). This water had a resistivity higher than 18 MΩ∙cm and a total organic carbon content below 6 ppm.

### 2.2. Preparation of Polyelectrolyte–Surfactant Mixtures

Two different protocols were employed for preparing polyelectrolyte–surfactant mixtures. The first protocol, referred to as *high-concentration mixing*, was adapted from the method previously developed by our group for preparing similar systems [36,37]. The procedure can be summarised as follows. First, the required volume of a QHECE stock aqueous solution (10 g/L) at pH = 6.5 and 25 °C to achieve a final mixture concentration of 5 g/L is measured and poured into a flask. Then, the required volume of a surfactant solution—prepared at a concentration of one order of magnitude higher than the target concentration in the final mixture—is added to the flask. Finally, the mixture is diluted with water to achieve the mixture with the required composition. The resulting mixtures were stirred overnight at 1000 rpm to ensure their complete homogenisation. The second mixing procedure, referred to as *gradient-free mixing*, is adapted from that proposed by Ravera et al. [38] for the preparation of particle–surfactant mixtures. This method relies on the preparation of the polyelectrolyte–surfactant by a 1:1 dilution. Specifically, a surfactant aqueous solution (pH = 6.5 and 25 °C) at twice the target concentration of the final mixture was dropwise added to a stirred aqueous solution of the polyelectrolyte (pH = 6.5 and 25 °C) with a concentration of 10 g/L to prepare a final mixture with a target concentration of QHECE of 5 g/L. This approach minimises the possible concentration gradients during mixing. The resulting mixture was stirred at 1000 rpm for 30 min to ensure uniformity and then left undisturbed overnight.

It should be noted that, for consistency and ease of comparison, all mixtures in this study were prepared by adding the surfactant solution to the QHECE solution. Nevertheless, considering the nonequilibrium character of polyelectrolyte–surfactant association [29], the order of addition is expected to significantly influence the association process. In fact, a reverse mixing order may could shift the appearance of mixtures with specific characteristics, such as turbidity peaks or charge neutralisation points, away from the predictions of equilibrium phase diagrams. These effects have been previously demonstrated in similar systems by Mészáros et al. [39], who highlighted the influence of kinetic trapping on complex morphology and phase behaviour. While an exploration of reversed mixing order is outside the scope of this study, it represents an important direction for future research.

### 2.3. Experimental Techniques

The turbidity of the mixtures, defined as *τ* = 1–10^−*A*^ with *A* being the absorbance of the analysed sample, was evaluated at a wavelength of 450 nm using a Jasco FP-6500 spectrophotometer (Jasco Inc., Tokyo, Japan). This specific wavelength was selected to ensure the absence of adsorption bands associated with the mixture components that interfere with the measurements. The effective charge density of the complexes was assessed through their electrophoretic mobility (*u*_e_) determined using Laser Doppler Velocimetry with a Nanosizer ZS (Malvern Instruments, Malvern, UK). The electrophoretic mobility and zeta potential (ζ) are related by Henry’s equation [40]. The specific conductance of the aqueous mixtures was assessed using a Metrohm 856 Conductometer (Metrohm AG, Herisau, Switzerland) fitted with a platinum 5-ring conductivity cell (Metrohm AG, Herisau, Switzerland). The results, representing the average of 10 separate measurements, demonstrated consistent accuracy and reproducibility exceeding 95%.

A QCM-Z500 quartz crystal microbalance with dissipation monitoring (QCM-D) from KSV Instruments Ltd. (Espoo, Finland), equipped with gold-coated AT-cut quartz crystals, was utilized to examine the adsorption behaviour of the polyelectrolyte–surfactant mixtures on negatively charged surfaces, mimicking the properties of damaged hair. The preparation of the quartz sensors involved surface modification by immersing them in Piranha solution (a 70% sulfuric acid and 30% hydrogen peroxide mixture) for 30 min, followed by extensive rinsing with Milli-Q water. A self-assembled monolayer of 3-mercapto-1-propane sulfonic acid (CAS No. 49594-30-1, Sigma-Aldrich, Saint Louis, MO, USA) was subsequently formed on the sensor surfaces, imparting a permanent negative charge to the gold substrate comparable to that of damaged hair fibres. The QCM-D technique was employed to measure the impedance spectrum of the quartz crystal at its fundamental frequency (*f*_0_ = 5 MHz) and odd harmonics up to the 11th overtone (central frequency, *f*_11_ = 55 MHz). Data analysis, based on the single-layer model proposed by Voinova et al. [41], linked the changes in resonance frequency (Δ*f*) and dissipation factor (Δ*D*) across various overtones to physical properties such as layer thickness, density, elasticity, and viscosity. Detailed descriptions of the analytical procedure can be found in prior work [42]. Importantly, incorporating the viscoelastic nature of the adsorbed polymer–surfactant film into QCM-D modelling is critical, as rigid boundary conditions do not accurately represent the adsorption behaviour of monolayers of these systems. These layers often exhibit heterogeneous surface coverage, as highlighted in extensive literature [37,43]. Furthermore, the significant hydration typical of this type of layer introduces dynamic components to the film that require viscoelastic modelling to account for viscous delay in the film’s mechanical response [44,45,46]. The QCM-D experiments were conducted in three stages. Initially, a stable signal for the oscillation of the quartz sensor was established by immersing it in the solvent (Milli-Q water at pH 6.5 and 25 °C). A stable signal was considered achieved when the changes in resonance frequency and dissipation factor were smaller than ±0.1 Hz and ±0.05 ppm, respectively, across the evaluated overtones. Following this, the polyelectrolyte–surfactant aqueous solution (prepared at pH 6.5 and 25 °C) was introduced into the measurement chamber. The injected volume was five times the chamber volume to ensure the complete replacement of the solvent with the polyelectrolyte–surfactant solution. This step led to a decrease in the resonance frequency and an increase in the dissipation factor, indicative of the formation of an adsorbed layer on the quartz sensor surface. Adsorption was monitored until a steady state was reached, as evidenced by the frequency and dissipation factor changes similar to those observed during baseline stabilisation. Once the adsorption equilibrium was achieved, the polyelectrolyte–surfactant solution in the chamber was replaced with the solvent (Milli-Q water at pH 6.5 and 25 °C) to ensure the complete removal of the polyelectrolyte–surfactant solution. During this final rinsing step, the system was allowed to equilibrate again, and the final thickness of the deposited layer was determined once the signal stabilised.

## 3. Results

### 3.1. Phase Behavior: A Nonequilibrium Issue

**Figure 2:**
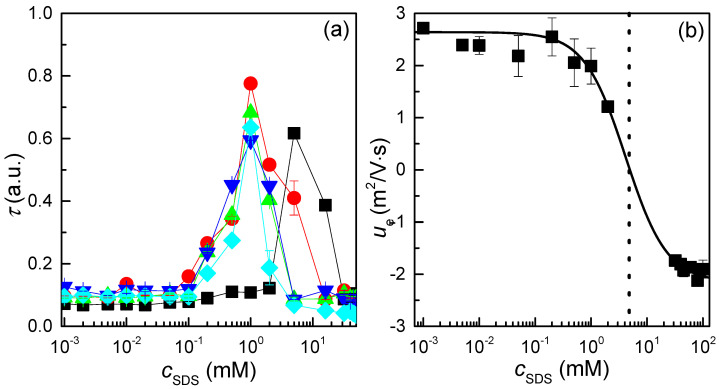
(**a**) Dependencies of the turbidity measured at 450 nm on *c*_SDS_ for QHECE–surfactant mixtures at different times after the mixture preparation. The results correspond to mixtures prepared using a *high-concentration mixing* approach. Symbols are referred to the experimental data obtained for different ageing times and the lines are guides by the eyes: (■) Fresh samples, (●) 6 days, (▲) 9 days, (▼) 16 days, and (♦) 21 days. Each datum corresponds to the average of 5 independent measurements with a deviation smaller than the symbol size. (**b**) The dependence of the electrophoretic mobility on *c*_SDS_ for the QHECE–surfactant. The results correspond to mixtures prepared using a *high-concentration mixing* approach at the end of the ageing. Symbols refer to the experimental data, the solid is guided by the eyes and the dashed vertical line indicates the charge neutralisation concentration. The data correspond to the average of 10 independent measurements, and the error bar indicates the standard deviation.

The phase behaviour of QHECE–SDS mixtures, with a fixed polyelectrolyte concentration of 5 g/L and increasing surfactant concentrations, was initially evaluated by measuring the turbidity of the mixtures. This was performed through absorbance measurements in the visible region, specifically at a wavelength (*λ*) of 450 nm. The choice of these conditions is not arbitrary but is based on the transparency of the mixture’s components to visible radiation in this spectral region. Thus, any change in absorbance in the visible region can be attributed to an increase in the sample’s turbidity, resulting from the formation of supramolecular aggregates in aqueous medium. Figure 2a illustrates the dependence of turbidity for mixtures prepared by a *high-concentration mixing* protocol, measured at different times after the preparation of the mixtures, on the surfactant concentration (*c*_SDS_) for mixtures with a fixed polymer concentration equivalent to 5 g/L.

The turbidity measurements give significant insights into the relationship between the polyelectrolyte and surfactant under the specific conditions employed to prepare the mixtures. First, irrespective of the ageing time, distinct regions with different characteristics are evident. At the lowest and highest surfactant concentrations, the turbidity remains almost constant at very low values, indicating that the samples are transparent. This behaviour is consistent with the presence of polyelectrolyte complexes carrying an excess charge, which is positive when the concentration of charged monomers exceeds that of SDS, or negative at the highest surfactant concentrations (see Figure 2b). In contrast, between these two regions of low turbidity, a sharp increase in the turbidity is observed, reaching a maximum before decreasing again as the surfactant concentration increases. This phenomenon can be attributed to the instability of the samples, which is likely a consequence of the reduced colloidal stability of the formed complexes due to their diminished charge. Over time, these complexes may undergo aggregation and sedimentation, resulting in the formation of a dense solid phase. Furthermore, the region corresponding to the turbidity peaks is relatively broad, spanning approximately one order of magnitude in surfactant concentration, which agrees with the findings of previous studies on QHECE–SDS mixtures [47,48,49,50].

More intriguing than the appearance of single-phase and biphasic regions in the phase behaviour of QHECE–SDS mixtures is the evolution of the turbidity curve with sample ageing. This evolution underscores the pivotal role of nonequilibrium factors in the mixing process. The complexes formed during mixing are not in a true equilibrium state, but they represent kinetically trapped aggregates [28,29,39]. This behaviour aligns with earlier studies that indicate that this mixing procedure promotes the formation of kinetically trapped aggregates, likely driven by Marangoni stresses occurring during the process [37]. These Marangoni stresses occur as a result of the existence of heterogeneities in concentration of the components within the aqueous medium during the mixing process. This concentration gradient within the mixtures provokes surface tension gradients that drive fluid motion. In the particular case of the *high-concentration mixing* approach, Marangoni stresses play a significant role in influencing the nonequilibrium assembly of complexes as surfactant is introduced rapidly into the QHECE solution. These stresses may induce localised flow patterns that lead to the uneven distribution of surfactant molecules and hinder the uniform association of QHECE with SDS, and consequently the formation of kinetically trapped aggregates. This is demonstrated by the observation that, in freshly prepared mixtures, the turbidity maximum occurs at an SDS concentration of approximately 6 mM. However, after ageing, this maximum shifts towards lower concentrations of the surfactant, stabilising at approximately 1 mM after two weeks. This indicates that the system is evolving towards equilibrium. During this process, the turbidity of certain samples initially appearing with the region of the turbidity peak gradually decreases to values compatible with a single-phase region. This behaviour can be attributed to the sedimentation of aggregates, as turbidity measurements only reflect the properties of the liquid phase. The sedimentation is the result of a lack of colloidal stability of the aggregates.

Additional insights into the association process between QHECE and SDS can be gained by analysing the dependence of electrophoretic mobility on SDS concentration for mixtures prepared via *high-concentration mixing* at the end of the ageing, as shown in the bottom panel of Figure 2b. These results agree with the above discussion related to the different regions in the phase behaviour in relation to the sign of the effective charge of the QHECE–SDS complexes. At low surfactant concentrations, QHECE–SDS complexes exhibit a positive effective charge, as evidenced by the positive values of their electrophoretic mobility. This behaviour can be attributed to an excess of charged monomers in relation to the surfactant concentration, which corresponds to a single-phase compositional region in the phase diagram. In this region, electrostatic attraction dominates the SDS binding to the QHECE, resulting in the progressive charge neutralisation of the complexes. As the concentration of the surfactant increases, the system undergoes a process of charge neutralisation followed by charge inversion. The latter results in the formation of complexes with a negative effective charge. As the neutralisation point is closer, the complexes become colloidally unstable, resulting in phase separation. This can be explained by considering that, in this region of intermediate concentrations, hydrophobic interactions among surfactant tails become significant, leading to the formation of larger aggregates and phase-separated structures, as evidenced by the appearance of a peak in the turbidity measurements. Conversely, at higher surfactant concentrations, the complexes are resolubilised due to the excess of surfactant units compared to the number of monomers. This results in the formation of overcharged complexes, which are characterised by the binding of SDS micelles to the QHECE chains. These complexes are overcompensated, acquiring a pronounced negative effective charge and highly hydrophilic. These observations are consistent with the findings by Li et al. on QHECE–SDS mixtures [50].

**Figure 3:**
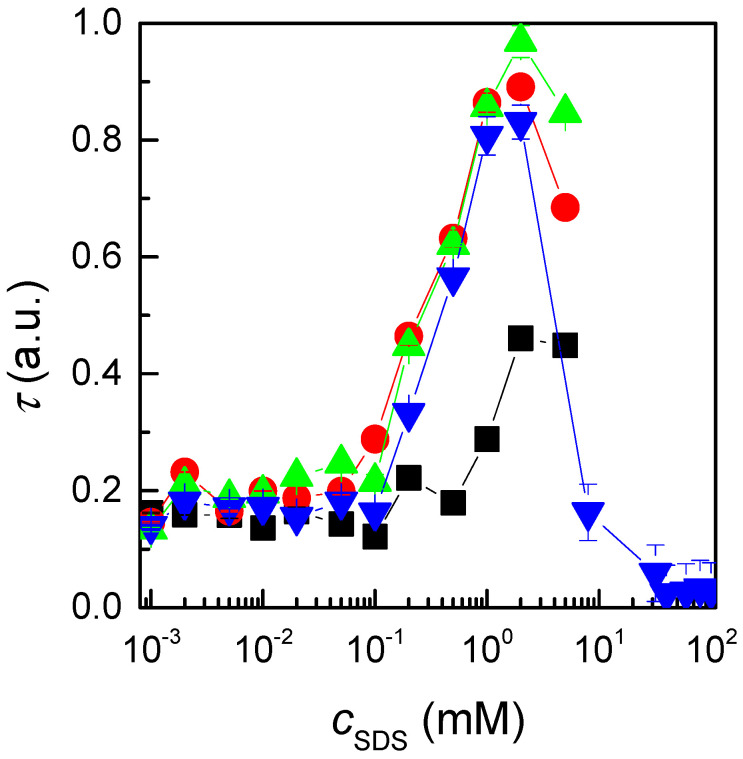
Dependences of the turbidity measured at 450 nm on *c*_SDS_ for QHECE–surfactant mixtures at different times after the mixture preparation. The results correspond to mixtures prepared using a *gradient-free mixing* approach. Symbols refer to the experimental data obtained for different ageing times and the lines are guides by the eyes: (■) Fresh samples, (●) 12 days, (▲) 14 days, and (▼) 18 days. The data correspond to the average of 5 independent measurements. Notice that for the sake of comparison with the results obtained for samples prepared by *high-concentration mixing*, the concentration range studied for samples with the larger ageing time was extended.

The above results clearly demonstrate the impact of nonequilibrium effects on the assembly of polyelectrolyte–surfactant complexes formed through *high-concentration mixing*. Nevertheless, a lack of clarity remains regarding the extent and implications of nonequilibrium phenomena in the association process. The initial key question concerns the role of Marangoni stresses during the mixing process. To gain further insight, it would be beneficial to investigate the preparation of QHECE–SDS mixtures using an alternative methodology, such as *gradient-free mixing*, which would minimise the contribution of concentration gradients during the mixing process. This approach can facilitate an understanding of the importance of Marangoni gradients in the assembly mechanism and their influence on the formation dynamics of the complexes. The second question concerns the relationship between the mixing methodology and the proximity of the resulting system to true equilibrium conditions. Therefore, it would be beneficial to determine whether a comparable steady state can be attained through the utilisation of distinct mixing approaches, or whether the resulting situation emerges as different. If the latter is the case, it would indicate that the nonequilibrium nature of the process has a significant impact on the ultimate structural and functional characteristics of the complexes. To address these questions, Figure 3 illustrates the dependence of turbidity on an SDS concentration for mixtures prepared using the *gradient-free mixing* approach.

In samples prepared using the *gradient-free mixing* approach, the absence of equilibrium is also evident in the initial stages following mixing. Nevertheless, the impact of Marangoni stresses is less pronounced in these mixtures in comparison to those prepared by *high-concentration mixing*, leading to a more gradual and uniform assembly process. This is evidenced by the fact that the *c*_SDS_ corresponding to the maximum undergoes a smaller shift from the fresh to the aged samples. Consequently, equilibrium is attained significantly faster, with an ageing time of less than two weeks. From an industrial perspective, this emphasises the importance of considering the mixing approach during the formulation processes. The evolution towards equilibrium may result in products exhibiting properties that deviate from the desired specifications due to its initial nonequilibrium nature. Therefore, the evaluation of ageing effects is essential to ensure the consistency and reliability of formulated products over time. Such assessments are crucial to mitigate the risks of performance variability and to optimise the design of formulations that meet application the specific requirements.

### 3.2. The Steady State Situation: True Equilibrium?

**Figure 4:**
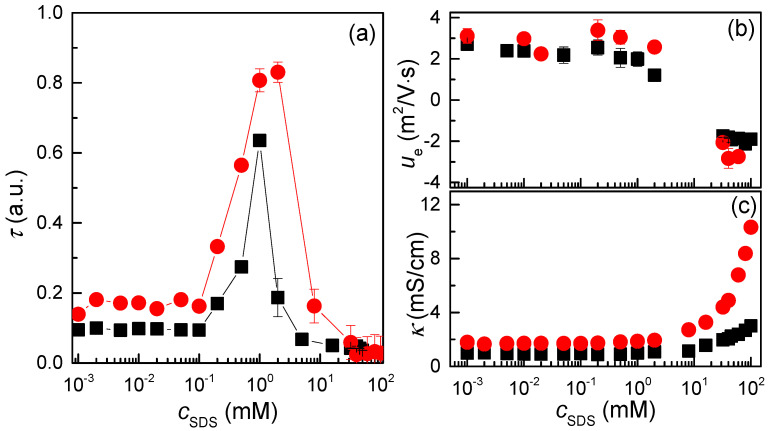
(**a**) Dependencies of the turbidity measured at 450 nm on *c*_SDS_ for the QHECE–surfactant mixtures prepared for both methods at the end of the ageing. The data correspond to the average of 5 independent measurements. (**b**) Dependence of the electrophoretic mobility on *c*_SDS_ for QHECE–surfactant mixtures prepared for both methods at the end of the ageing. The data correspond to the average of 10 independent measurements, and the error bar indicates the standard deviation. (**c**) Dependence of the specific conductance on *c*_SDS_ for QHECE–surfactant mixtures prepared for both methods at the end of ageing. The data correspond to the average of 10 independent measurements, and the error bar indicates the standard deviation. In all the panels: Symbol refer to the experimental data and lines, and where these are included, are guides for the eyes. The results show a comparison between the results corresponding to samples at the end of the ageing prepared by *high-concentration mixing* (■) and *gradient-free mixing* (●).

As previously discussed, one of the key unresolved questions concerns the similarities and differences between samples prepared using different mixing procedures once they have reached a steady state. To investigate this, the turbidity dependence on SDS concentrations for aged samples prepared using both explored methods is presented in Figure 4a. This comparison aims to elucidate whether the final properties of the complexes are influenced solely by the equilibrium conditions or retain a signature of the specific mixing process employed. Such insights are crucial for comprehending the extent to which the mixing protocol influences the final state of the polyelectrolyte–surfactant complexes.

The results provide a clear visual representation of the way in which the mixing methodology affects turbidity profiles, which serve as an indirect indicator of the characteristics of the complexes. The analysis of the data indicates that, while the concentration dependence of the curves is qualitatively similar for both mixing methods, there are notable differences in the characteristics of the resulting complexes. In particular, at all concentrations, samples prepared by *gradient-free mixing* display higher turbidity values than those prepared by *high-concentration mixing*. This calls for a re-evaluation of the assumption that the steady-state condition obtained after ageing necessarily corresponds to the true equilibrium situation. This emphasises the complexity of defining true equilibrium conditions in polyelectrolyte–surfactant systems. The observed differences indicate that the steady state achieved is not only dependent on the thermodynamic parameters but also intrinsically linked to the kinetics and dynamics of the assembly process. This has significant implications for the design and optimisation of formulations, particularly in applications where specific structural or functional properties are critical. For example, it must be recalled that, for ensuring a good performance of polyelectrolyte–surfactant complexes in shampoo or hair conditioner formulations, a thick adsorbed layer on the hair fibre is important. The theta condition is achieved on the coacervation region, i.e., high turbidity values [51]; thus, the different areas of the turbidity vs. *c*_SDS_ curves suggest the different adsorption of the complexes onto the hair surfaces.

In light of the aforementioned observations, an interesting question arises as to whether the mixing procedure affects the effective charge of the complexes. Based on the similarities in phase behaviour inferred from the turbidity data, it can be hypothesised that the mixing method may not significantly affect the net charge of the complexes. Nevertheless, further investigation is required to gain a full understanding of the interplay between mixing dynamics and charge characteristics. To address this question, the electrophoretic mobility of the complexes was analysed for samples prepared using different mixing procedures, as this provides a direct measure of their effective charge. A comparison of the data presented in Figure 4b provides a more detailed insight into whether the turbidity profiles are mirrored in the charge properties of the complexes.

The electrophoretic mobility data, in a manner analogous to the turbidity measurements, evidence a qualitatively similar concentration-dependent profile regardless of the mixing procedure employed to prepare the mixtures. In all cases, the complexes formed via *gradient-free mixing* exhibit a slightly higher effective charge compared to those obtained through *high-concentration mixing*. Nevertheless, the extent of this difference is insufficient to justify a physical sound evaluation. It can therefore be assumed that the effective charge of the complexes remains fundamentally unchanged regardless of the mixing methodology employed. Thus, it is plausible that the observed differences between the steady state samples must be attributable to variations in the arrangement of the polymer and surfactant chains, rather than intrinsic alterations in the true charge of the complexes. This finding corroborates the hypothesis that the mixing procedure primarily affects physical properties, such as aggregation behaviour and turbidity, while leaving the electrostatic characteristics of the complexes largely unaltered, even though subtle differences on the charge can be observed.

Further insight into the influence of nonequilibrium dynamics during the mixing process can be gained by examining the specific conductance (κ) of the polyelectrolyte–surfactant mixtures prepared using the two different mixing approaches (see Figure 4c). As for the turbidity measurements, the results show qualitatively similar concentration dependencies regardless of the mixing method used. In both cases, the conductivity remains relatively constant with an increasing SDS concentration of up to about 10 mM. Beyond this threshold, a sharp increase in conductivity is observed as the surfactant concentration continues to increase. This behaviour mirrors that observed for ionic surfactants in solution, where a sharp increase in conductivity is typically associated with the formation of micelles. The onset of this sharp increase suggests the presence of an apparent critical micellar concentration (CMC), which occurs above the CMC of pure SDS and marks the transition from the associative behaviour of the complexes to the formation of free surfactant micelles in the solution [52]. On the other hand, significant differences in the absolute values of conductivity were observed, highlighting the impact of the mixing procedure on the final characteristics of the samples. These findings further underscore the critical role of mixing dynamics in shaping the properties of the resulting polyelectrolyte–surfactant complexes. Specifically, the experimental results demonstrate that samples prepared via the *gradient-free mixing* method exhibit higher specific conductivity compared to those prepared via *high-concentration mixing*. This difference can be attributed to variations in ion availability and the degree of micelle formation during the mixing process. A plausible explanation lies in the fact that the *high-concentration mixing* method introduces surfactant at concentrations exceeding the critical micelle concentration (CMC) early in the process. This likely facilitates micelle formation and the development of kinetically trapped aggregates, which may encapsulate counterions. Such encapsulation reduces ion mobility and, consequently, their contribution to the overall conductivity. In contrast, the *gradient-free mixing* method promotes a more gradual association between QHECE and SDS, minimising the formation of kinetically trapped aggregates and ensuring a higher availability of free ions in solution. This may be in agreement with the formation of turbid samples at the highest SDS concentrations during the initial stages of ageing for samples prepared using the *high-concentration mixing* method. Additionally, differences in the mixing processes could be also influenced by the viscosity of the resulting mixtures, further affecting ion mobility. It is worth noting that the role of Marangoni stresses during association cannot be neglected to provide an explanation of the different values of the specific conductance. In *high-concentration mixing*, the rapid introduction of surfactant can create significant concentration gradients, generating Marangoni stresses that may cause local ion depletion in certain regions of the solution. This effect could further contribute to the lower conductivity observed in *high-concentration mixing* samples, compared to *gradient-free mixing* one, where such stresses are minimised.

### 3.3. Adsorption of the QHECE–SDS Complexes on Negatively Charged Surfaces

**Figure 5:**
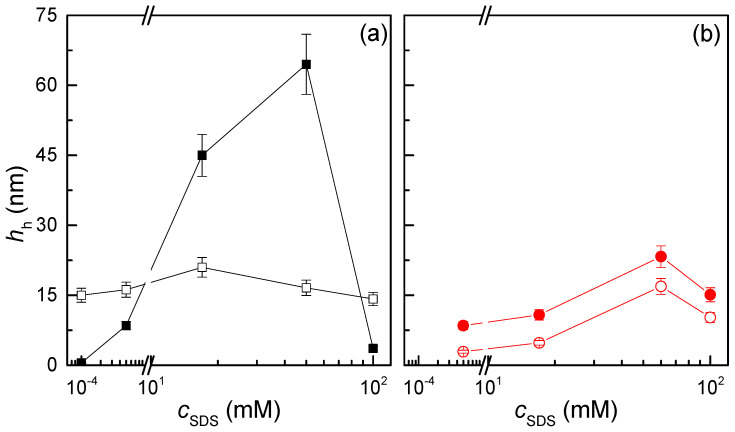
Dependence of the hydrodynamic thickness, *h*_h_, on the SDS concentration for the adsorption of the QHECE–SDS complex on negatively charged solid surfaces. (**a**) Results corresponding to the adsorption of samples prepared by *high-concentration mixing*. (**b**) Results corresponding to the adsorption of samples prepared by *gradient-free mixing*. Open and solid symbols represent the data obtained before rinsing and after rinsing, respectively.

It is expected that the adsorption of QHECE–SDS complexes onto solid surfaces will be significantly influenced by the complex bulk behaviour of these systems [16,53,54,55]. This section is devoted to the analysis of polyelectrolyte–surfactant films deposited onto negatively charged surfaces, with a particular focus on the characterisation of the adsorbed layers. To quantify this, we employ quartz crystal microbalance with dissipation (QCM-D) experiments, which permit precise measurement of the hydrodynamic thickness (*h*_h_) of the adsorbed films. Figure 5 displays the dependence on the SDS concentration of quantity of material adsorbed, represented as the hydrodynamic thickness both after the deposition and upon rinsing with pure water for samples prepared by the two different approaches.

The results show the existence of different regions in the effect of rinsing as a function of the concentration of surfactant. At the lowest SDS concentrations, it is observed that the rinsing process leads to a clear decrease in the adsorbed amount. This can be rationalised by considering that, in this compositional region, the rinsing with water results in the removal of molecules that are not strongly adsorbed on the surface, and thus exclusively leaving attached the remaining complexes that are adsorbed through electrostatic interactions. At intermediate concentrations, corresponding with the turbidity peak, the situation changes significantly, with the rinsing resulting in an increase in the hydrodynamic thickness of the layer. This counterintuitive effect may be interpreted as the result of a precipitation phenomenon induced by dilution [7,56,57,58,59,60]. Thus, the dilution process of concentrated mixtures, occurring during rinsing, can take the system to a compositional region where the precipitation of complexes is favoured. This mechanism, involved in the performance of most of traditional shampoos, justifies the increase in the layer thickness upon rinsing with water for intermediate concentrations of surfactants. At the highest surfactant concentrations, the behaviour returns to a situation similar to that found for the lowest surfactant concentration. This can be understood considering that the dilution process for such high surfactant concentrations maintains the system far from the boundaries of the phase separation, and therefore, the rinsing only contributes to remove weakly adsorbed chains. It is worth mentioning that a qualitatively similar behaviour was observed for the samples prepared following both preparation approaches.

**Figure 6:**
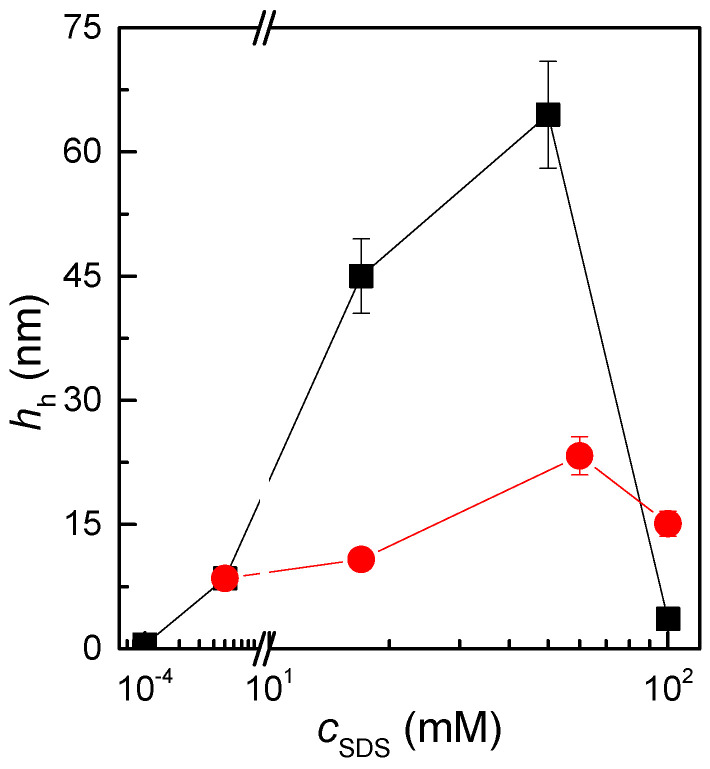
Dependence of the hydrodynamic thickness, *h*_h_, on the SDS concentration for the adsorption of QHECE–SDS co0mplex on negatively charged solid surfaces. The results show a comparison between the results corresponding to aged samples prepared by *high-concentration mixing* (■) and *gradient-free mixing* (●).

From the application perspective, it is more interesting to analyse the layer thickness after rinsing, as shown in Figure 6. These results evidence the presence of three regions in the adsorption, which can be associated with the phase behaviour discussed above. At low SDS concentrations, where single-phase systems are formed, the adsorption is relatively low. This can be explained by considering that, for such concentration ranges, the complexes are strongly hydrophilic and remain well dispersed in the aqueous solution. This leads to a situation which is characterised by a reduced depletion from the solution to the surface. However, a certain degree of adsorption occurs as result of the electrostatic interactions. At intermediate surfactant concentrations, a strong adsorption occurs. This can be understood if we consider that, in this region, the system enters in a phase-separation region. Across this region, the complexes can be depleted from the aqueous medium and deposited onto the solid surfaces, significantly increasing the deposition. On the contrary, at the highest surfactant concentrations, the adsorption is again worsened. This is easily understood by considering that, when the SDS concentration is relatively high, the complexes become hydrophilic again and remain dispersed in the aqueous medium without any significant trend to be depleted onto the surface. Moreover, the complexes assume a negative charge which introduces a repulsive interaction with the surface.

The comparison of the results obtained for the deposition of samples prepared for both methods also evidences the effect of nonequilibrium observed in the solution of the aqueous dispersions of the QHECE–SDS mixtures. The more significant difference appears in the adsorbed amount, which is more than three times higher for samples prepared by *high-concentration mixing*. This may be correlated to the different turbidity of the samples prepared for each method. In the case of the samples prepared by *gradient-free mixing*, the values of turbidity are higher than when the mixtures are prepared by *high-concentration mixing*, which suggests that even though the system is within a region characterised by the formation of solid particles, these are slightly more stable in the aqueous medium. This is also supported by the slightly higher absolute values of effective charge evidenced by electrophoretic mobility measurements. Thus, their depletion from the solution is less important, and therefore the adsorption is reduced.

## 4. Conclusions

This study provides insights into the behaviour of quaternized hydroxyethylcellulose ethoxylate (QHECE)–sodium dodecyl sulphate (SDS) complexes, focusing on their phase behaviour, adsorption properties, and assembly on negatively charged substrates. The obtained results strongly suggest that the quaternized groups act as the primary binding sites for SDS molecules to QHECE. The charge neutralisation and subsequent charge inversion observed in electrophoretic mobility measurements further confirm the involvement of the quaternized cationic sites in SDS binding. Moreover, the findings demonstrate that the preparation method plays a crucial role in determining the final steady-state and final properties of the QHECE–SDS complexes. Both *high-concentration* and *gradient-free mixing* approaches revealed nonequilibrium dynamics during complex formation, as evidenced by turbidity, electrophoretic mobility, and conductivity measurements. These dynamics affect the structural and functional attributes of the complexes, particularly their charge, aggregation behaviour, and stability. In fact, nonequilibrium processes, including Marangoni stresses during mixing, lead to kinetically trapped aggregates that evolve towards a steady-state time. The influence of ageing of the complexes emphasises the need to consider storage conditions and preparation timelines when designing formulations. *Gradient-free mixing*, which minimises concentration gradients, was found to reduce the impact of nonequilibrium effects and facilitate the reaching of steady-state conditions compared to *high-concentration mixing*. The results also highlight the importance of intermediate SDS concentrations, which promote phase separation and enhanced adsorption due to favourable electrostatic and hydrophobic interactions.

This study underscores the critical role of adsorption in the performance of QHECE-based formulations, particularly in applications like hair care, where deposition on negatively charged surfaces is vital. Quartz crystal microbalance with dissipation monitoring (QCM-D) experiments confirmed that adsorption is strongly influenced by the bulk properties of the QHECE–SDS mixtures, including their aggregation state and conditions. The observed differences between *high-concentration* and *gradient-free mixing* suggest that the preparation protocol not only impacts the time required to reach equilibrium but also the characteristics of the resulting complexes. This has practical implications for industrial formulation processes, as the choice of mixing method can significantly affect product performance and stability over time. Additionally, the results demonstrate the interplay between phase behaviour and adsorption. At low SDS concentrations, the complexes remain highly hydrophilic, leading to low surface deposition. In contrast, at intermediate concentrations, phase separation occurs, resulting in robust adsorption due to the depletion of aggregates from the bulk and their deposition onto negatively charged surfaces. At higher concentrations, the complexes again present their hydrophilic nature and exhibit reduced adsorption due to electrostatic repulsion. This understanding of the concentration-dependent behaviour of QHECE–SDS complexes provides valuable guidelines for optimising formulations.

From an application perspective, these findings are particularly relevant for the development of hair care products, where the deposition of conditioning agents on damaged hair is a critical performance criterion. The use of model substrates to mimic the charge density and surface properties of damaged hair provided a controlled environment to study these interactions, offering insights that can be directly translated into practical applications. The results demonstrate that formulations with intermediate SDS concentrations are likely to exhibit optimal deposition and performance, highlighting the importance of fine-tuning surfactant concentrations in cosmetic products.

In conclusion, this research advances the understanding of polyelectrolyte–surfactant interactions and their implications for industrial applications. The findings underscore the importance of considering mixing protocols, ageing effects, and phase behaviour in the design of stable and effective formulations. By elucidating the complex interplay between bulk and interfacial properties, this study provides a foundation for developing QHECE-based products with enhanced performance and stability.

## Data Availability

Data are available upon request.

## References

[1] Li Y., Chen C., Cui G., Liu L., Zhou C., Wu G. (2024). Hydroxyethyl Cellulose-Based Stretchable, Antifreeze, Ion-Conductive Hydrogel Sensor. Eur. Polym. J..

[2] Pan Y., Wang J., Yang S., Fu J., Lebongo Eteme Y. (2023). Research Progress of Hydroxyethyl Cellulose Materials in Oil and Gas Drilling and Production. Cellulose.

[3] Rukmanikrishnan B., Ramalingam S., Rajasekharan S.K., Lee J., Lee J. (2020). Binary and Ternary Sustainable Composites of Gellan Gum, Hydroxyethyl Cellulose and Lignin for Food Packaging Applications: Biocompatibility, Antioxidant Activity, UV and Water Barrier Properties. Int. J. Biol. Macromol..

[4] Noreen A., Zia K.M., Tabasum S., Khalid S., Shareef R. (2020). A Review on Grafting of Hydroxyethylcellulose for Versatile Applications. Int. J. Biol. Macromol..

[5] EL-Haddad M.N. (2014). Hydroxyethylcellulose Used as an Eco-Friendly Inhibitor for 1018 c-Steel Corrosion in 3.5% NaCl Solution. Carbohydr. Polym..

[6] Guzmán E., Ortega F., Baghdadli N., Luengo G.S., Rubio R.G. (2011). Effect of the Molecular Structure on the Adsorption of Conditioning Polyelectrolytes on Solid Substrates. Colloids Surf. A.

[7] Fernández-Peña L., Guzmán E., Oñate-Martínez T., Fernández-Pérez C., Ortega F., Rubio R.G., Luengo G.S. (2023). Dilution-Induced Deposition of Concentrated Binary Mixtures of Cationic Polysaccharides and Surfactants. Polymers.

[8] Carvalho J.P., Martins M., Cavaco-Paulo A. (2024). Cellulose Formulations for Hair Modelling. Cellulose.

[9] Jilal I., El Barkany S., Bahari Z., Sundman O., El Idrissi A., Abou-Salama M., Romane A., Zannagui C., Amhamdi H. (2018). New Quaternized Cellulose Based on Hydroxyethyl Cellulose (HEC) Grafted EDTA: Synthesis, Characterization and Application for Pb (II) and Cu (II) Removal. Carbohydr. Polym..

[10] Wang H., Qin Z., Zhang Y., Liu D., Cao Y. (2023). Complexation between Poly (Styrene-Co-Methacrylic Acid) and Polyquaternium for Use in Shampoo Formulations. J. Mol. Liq..

[11] Guzmán E., Ortega F., Baghdadli N., Cazeneuve C., Luengo G.S., Rubio R.G. (2011). Adsorption of Conditioning Polymers on Solid Substrates with Different Charge Density. ACS Appl. Mater. Interfaces.

[12] Zhou Z., Xu J., Zhu S., Wang B., Li J., Ying G., Chen K. (2024). A Gentle Conditioning Agent Consisted of Oppositely-Charged-Induced Cellulose Nanocrystal and Cationic Cellulose: Stability, Conditioning and Delivery. J. Clean. Prod..

[13] Zhou G., Wang Q., Li S., Huang Q., Liu Z. (2023). Effect of a Newly Synthesized Anionic Gemini Surfactant Composite Fracturing System on the Wettability of Coking Coal. Process Saf. Environ. Prot..

[14] Abou-alfitooh S.A.M., El-hoshoudy A.N. (2024). Eco-Friendly Modified Biopolymers for Enhancing Oil Production: A Review. J. Polym. Environ..

[15] Wang T., Ye J. (2023). Rheological and Fracturing Characteristics of a Cationic Guar Gum. Int. J. Biol. Macromol..

[16] Lindman B., Antunes F., Aidarova S., Miguel M., Nylander T. (2014). Polyelectrolyte-Surfactant Association—From Fundamentals to Applications. Colloid J..

[17] Gradzielski M. (2023). Polymer–Surfactant Interaction for Controlling the Rheological Properties of Aqueous Surfactant Solutions. Curr. Opin. Colloid Interface Sci..

[18] Luengo G.S., Leonforte F., Greaves A., Rubio R.G., Guzman E. (2023). Physico-Chemical Challenges on the Self-Assembly of Natural and Bio-Based Ingredients on Hair Surfaces: Towards Sustainable Haircare Formulations. Green Chem..

[19] Guzmán E., Ortega F., Prolongo M.G., Starov V.M., Rubio R.G. (2011). Influence of the Molecular Architecture on the Adsorption onto Solid Surfaces: Comb-like Polymers. Phys. Chem. Chem. Phys..

[20] Breakspear S., Smith J.R., Luengo G. (2005). Effect of the Covalently Linked Fatty Acid 18-MEA on the Nanotribology of Hair’s Outermost Surface. J. Struct. Biol..

[21] Korte M., Akari S., Kühn H., Baghdadli N., Möhwald H., Luengo G.S. (2014). Distribution and Localization of Hydrophobic and Ionic Chemical Groups at the Surface of Bleached Human Hair Fibers. Langmuir.

[22] Robbins C.R. (2012). Chemical and Physical Behavior of Human Hair.

[23] Baghdadli N., Luengo G.S., Recherche L. (2008). A Closer Look at the Complex Hydrophilic/Hydrophobic Interactions Forces at the Human Hair Surface. J. Phys. Conf. Ser..

[24] Xiong W., Zeng Z., Li X., Zeng G., Xiao R., Yang Z., Xu H., Chen H., Cao J., Zhou C. (2019). Ni-Doped MIL-53(Fe) Nanoparticles for Optimized Doxycycline Removal by Using Response Surface Methodology from Aqueous Solution. Chemosphere.

[25] Xiong W., Zeng G., Yang Z., Zhou Y., Zhang C., Cheng M., Liu Y., Hu L., Wan J., Zhou C. (2018). Adsorption of Tetracycline Antibiotics from Aqueous Solutions on Nanocomposite Multi-Walled Carbon Nanotube Functionalized MIL-53(Fe) as New Adsorbent. Sci. Total Environ..

[26] Cao J., Xu B., Lin H., Luo B., Chen S. (2012). Chemical Etching Preparation of BiOI/BiOBr Heterostructures with Enhanced Photocatalytic Properties for Organic Dye Removal. Chem. Eng. J..

[27] Bain C.D., Claesson P.M., Langevin D., Meszaros R., Nylander T., Stubenrauch C., Titmuss S., von Klitzing R. (2010). Complexes of Surfactants with Oppositely Charged Polymers at Surfaces and in Bulk. Adv. Colloid Interface Sci..

[28] Naderi A., Claesson P.M., Bergström M., Dėdinaitė A. (2005). Trapped Non-Equilibrium States in Aqueous Solutions of Oppositely Charged Polyelectrolytes and Surfactants: Effects of Mixing Protocol and Salt Concentration. Colloids Surf. A.

[29] Varga I., Campbell R.A. (2017). General Physical Description of the Behavior of Oppositely Charged Polyelectrolyte/Surfactant Mixtures at the Air/Water Interface. Langmuir.

[30] Puente-Santamaría A., Ortega F., Maestro A., Rubio R.G., Guzmán E. (2024). Non-Equilibrium States in Polyelectrolyte-Surfactant Systems at Fluid Interfaces: A Critical Review. Curr. Opin. Colloid Interface Sci..

[31] Fernández-Peña L., Abelenda-Nuñez I., Hernández-Rivas M., Ortega F., Rubio R.G., Guzmán E. (2020). Impact of the Bulk Aggregation on the Adsorption of Oppositely Charged Polyelectrolyte-Surfactant Mixtures onto Solid Surfaces. Adv. Colloid Interface Sci..

[32] Guzmán E., Fernández-Peña L., Ortega F., Rubio R.G. (2020). Equilibrium and Kinetically Trapped Aggregates in Polyelectrolyte–Oppositely Charged Surfactant Mixtures. Curr. Opin. Colloid Interface Sci..

[33] Guzmán E., Maestro A., Ortega F., Rubio R.G. (2023). Association of Oppositely Charged Polyelectrolyte and Surfactant in Solution: Equilibrium and Nonequilibrium Features. J. Phys. Cond. Matter.

[34] Ghasemi M., Jamadagni S.N., Johnson E.S., Larson R.G. (2023). A Molecular Thermodynamic Model of Coacervation in Solutions of Polycations and Oppositely Charged Micelles. Langmuir.

[35] Roa K., Boulett A., Oyarce E., Sánchez J. (2023). Removal of Cr(VI) by Ultrafiltration Enhanced by a Cellulose-Based Soluble Polymer. J. Water Proc. Eng..

[36] Akanno A., Guzmán E., Fernández-Peña L., Llamas S., Ortega F., Rubio R.G. (2018). Equilibration of a Polycation—Anionic Surfactant Mixture at the Water/Vapor Interface. Langmuir.

[37] Fernández-Peña L., Guzmán E., Leonforte F., Serrano-Pueyo A., Regulski K., Tournier-Couturier L., Ortega F., Rubio R.G., Luengo G.S. (2020). Effect of Molecular Structure of Eco-Friendly Glycolipid Biosurfactants on the Adsorption of Hair-Care Conditioning Polymers. Colloids Surf. B Biointerfaces.

[38] Ravera F., Santini E., Loglio G., Ferrari M., Liggieri L. (2006). Effect of Nanoparticles on the Interfacial Properties of Liquid/Liquid and Liquid/Air Surface Layers. J. Phys. Chem. B.

[39] Mészáros R., Thompson L., Bos M., Varga I., Gilányi T. (2003). Interaction of Sodium Dodecyl Sulfate with Polyethyleneimine: Surfactant-Induced Polymer Solution Colloid Dispersion Transition. Langmuir.

[40] Smoluchowski M. (1921). Handbuch Der Elektrizität Und Des Magnetismus.

[41] Voinova M.V., Rodahl M., Jonson M., Kasemo B. (1999). Viscoelastic Acoustic Response of Layered Polymer Films at Fluid-Solid Interfaces: Continuum Mechanics Approach. Phys. Script..

[42] Fernández-Peña L., Guzmán E., Ortega F., Bureau L., Leonforte F., Velasco D., Rubio R.G., Luengo G.S. (2021). Physico-Chemical Study of Polymer Mixtures Formed by a Polycation and a Zwitterionic Copolymer in Aqueous Solution and upon Adsorption onto Negatively Charged Surfaces. Polymer.

[43] Johannsmann D., Reviakine I., Richter R.P. (2009). Dissipation in Films of Adsorbed Nanospheres Studied by Quartz Crystal Microbalance (QCM). Anal. Chem..

[44] Konyalı E., Cengiz H.Y., Müftüler A., Deligöz H. (2023). Monitoring the Salt Stability and Solvent Swelling Behavior of PAH-based Polyelectrolyte Multilayers by Quartz Crystal Microbalance with Dissipation. Polym. Eng. Sci..

[45] Davantès A., Nigen M., Sanchez C., Renard D. (2023). Impact of Hydrophobic and Electrostatic Forces on the Adsorption of Acacia Gum on Oxide Surfaces Revealed by QCM-D. Colloids Interfaces.

[46] Reviakine I., Johannsmann D., Richter R.P. (2011). Hearing What You Cannot See and Visualizing What You Hear: Interpreting Quartz Crystal Microbalance Data from Solvated Interfaces. Anal. Chem..

[47] Regismond S.T.A., Winnik F.M., Goddard E.D. (1996). Surface Viscoelasticity in Mixed Polycation Anionic Surfactant Systems Studied by a Simple Test. Colloids Surf. A.

[48] Goddard E.D., Hannan R.B. (1977). Polymer/Surfactant Interactions. J. Am. Oil Chem. Soc..

[49] Goddard E.D., Hannan R.B. (1976). Cationic Polymer/Anionic Surfactant Interactions. J. Colloid Interface Sci..

[50] Li D., Kelkar M.S., Wagner N.J. (2012). Phase Behavior and Molecular Thermodynamics of Coacervation in Oppositely Charged Polyelectrolyte/Surfactant Systems: A Cationic Polymer JR 400 and Anionic Surfactant SDS Mixture. Langmuir.

[51] Neitzel A.E., Fang Y.N., Yu B., Rumyantsev A.M., de Pablo J.J., Tirrell M.V. (2021). Polyelectrolyte Complex Coacervation across a Broad Range of Charge Densities. Macromolecules.

[52] Kogej K., Škerjanc J. (1999). Fluorescence and Conductivity Studies of Polyelectrolyte-Induced Aggregation of Alkyltrimethylammonium Bromides. Langmuir.

[53] Svensson A.V., Johnson E.S., Nylander T., Piculell L. (2010). Surface Deposition and Phase Behavior of Oppositely Charged Polyion−Surfactant Ion Complexes. 2. A Means to Deliver Silicone Oil to Hydrophilic Surfaces. ACS Appl. Mater. Interfaces.

[54] Svensson A.V., Huang L., Johnson E.S., Nylander T., Piculell L. (2009). Surface Deposition and Phase Behavior of Oppositely Charged Polyion/Surfactant Ion Complexes. 1. Cationic Guar versus Cationic Hydroxyethylcellulose in Mixtures with Anionic Surfactants. ACS Appl. Mater. Interfaces.

[55] Clauzel M., Johnson E.S., Nylander T., Panandiker R.K., Sivik M.R., Piculell L. (2011). Surface Deposition and Phase Behavior of Oppositely Charged Polyion–Surfactant Ion Complexes. Delivery of Silicone Oil Emulsions to Hydrophobic and Hydrophilic Surfaces. ACS Appl. Mater. Interfaces.

[56] Puente-Santamaría A., Monge-Corredor J., Ortega F., Rubio R.G., Guzmán E. (2024). Dilution-Controlled Deposition of Mixtures of a Synthetic Polycation and a Natural Origin Polyelectrolyte with Anionic Surfactants on Negatively Charged Surfaces. Colloids Surf. A.

[57] Miyake M., Kakizawa Y. (2002). Study on the Interaction between Polyelectrolytes and Oppositely Charged Ionic Surfactants. Solubilized State of the Complexes in the Postprecipitation Region. Colloid Polym. Sci..

[58] Miyake M. (2017). Recent Progress of the Characterization of Oppositely Charged Polymer/Surfactant Complex in Dilution Deposition System. Adv. Colloid Interface Sci..

[59] Hössel P., Dieing R., Nörenberg R., Pfau A., Sander R. (2000). Conditioning Polymers in Today’s Shampoo Formulations—Efficacy, Mechanism and Test Methods. Int. J. Cosmet. Sci..

[60] Kakizawa Y., Miyake M. (2019). Creation of New Functions by Combination of Surfactant and Polymer—Complex Coacervation with Oppositely Charged Polymer and Surfactant for Shampoo and Body Wash. J. Oleo Sci..

